# Molecular Modeling for Structural Insights Concerning the Activation Mechanisms of F1174L and R1275Q Mutations on Anaplastic Lymphoma Kinase

**DOI:** 10.3390/molecules23071610

**Published:** 2018-07-02

**Authors:** Cheng-Han Jiang, Chong-Xian Huang, Ya-Jyun Chen, Yu-Chung Chuang, Bo-Yen Huang, Chia-Ning Yang

**Affiliations:** Department of Life Sciences, National University of Kaohsiung, Kaohsiung 811, Taiwan; selina571215@gmail.com (C.-H.J.); htchuang8514@gmail.com (C.-X.H.); joyce770109@yahoo.com.tw (Y.-J.C.); ilovefancier@gmail.com (Y.-C.C.); distortfact8@gmail.com (B.-Y.H.)

**Keywords:** ALK, mutation, molecular dynamic simulation

## Abstract

Anaplastic lymphoma kinase (ALK) is a receptor tyrosine kinase involved in various cancers. In its basal state, the structure of ALK is in an autoinhibitory form stabilized by its A-loop, which runs from the N-lobe to the C-lobe of the kinase. Specifically, the A-loop adopts an inhibitory pose with its proximal A-loop helix (αAL-helix) to anchor the αC-helix orientation in an inactive form in the N-lobe; the distal portion of the A-loop is packed against the C-lobe to block the peptide substrate from binding. Upon phosphorylation of the first A-loop tyrosine (Y1278), the αAL-helix unfolds; the distal A-loop detaches from the C-lobe and reveals the P+1 pocket that accommodates the residues immediately after their phosphorylation, and ALK is activated accordingly. Recently, two neuroblastoma mutants, F1174L and R1275Q, have been determined to cause ALK activation without phosphorylation on Y1278. Notably, F1174 is located on the C-terminus of the αC-helix and away from the A-loop, whereas R1275 sits on the αAL-helix. In this molecular modeling study, we investigated the structural impacts of F1174L and R1275Q that lead to the gain-of-function event. Wild-type ALK and ALK with phosphorylated Y1278 were also modeled for comparison. Our modeling suggests that the replacement of F1174 with a smaller residue, namely leucine, moves the αC-helix and αAL-helix into closer contact and further distorts the distal portion of the A-loop. In wild-type ALK, R1275 assumes the dual role of maintaining the αAL-helix–αC-helix interaction in an inactive form and securing αAL-helix conformation through the D1276–R1275 interaction. Accordingly, mutating R1275 to a glutamine reorients the αC-helix to an active form and deforms the entire A-loop. In both F1174L and R1275Q mutants, the A-loop rearranges itself to expose the P+1 pocket, and kinase activity resumes.

## 1. Introduction

Anaplastic lymphoma kinase (ALK) is a member of the superfamily of the insulin receptor protein tyrosine kinase; ALK participates in embryonic nervous system development during embryogenesis with decreased expression after birth [1]. Accumulating evidence indicates that dysregulation of ALK is associated with numerous diseases such as anaplastic large cell lymphomas [2], lung cancer [3] and neuroblastomas [4]. Full-length ALK consists of an extracellular portion responsible for ligand binding; a transmembrane segment; and an intracellular portion with a juxtamembrane (JM) segment, protein kinase domain and carboxy terminal tail. In its basal condition, the kinase domain of the ALK is inactive, but can be activated through binding with an activating ligand such as midkine [5] or pleiotrophin [6] at the extracellular portion. Ligand binding induces ALK dimerization, resulting in the transphorylation of Y1278 on the activation loop (A-loop) by the partner ALK protein kinase domain [7].

**Figure 1:**
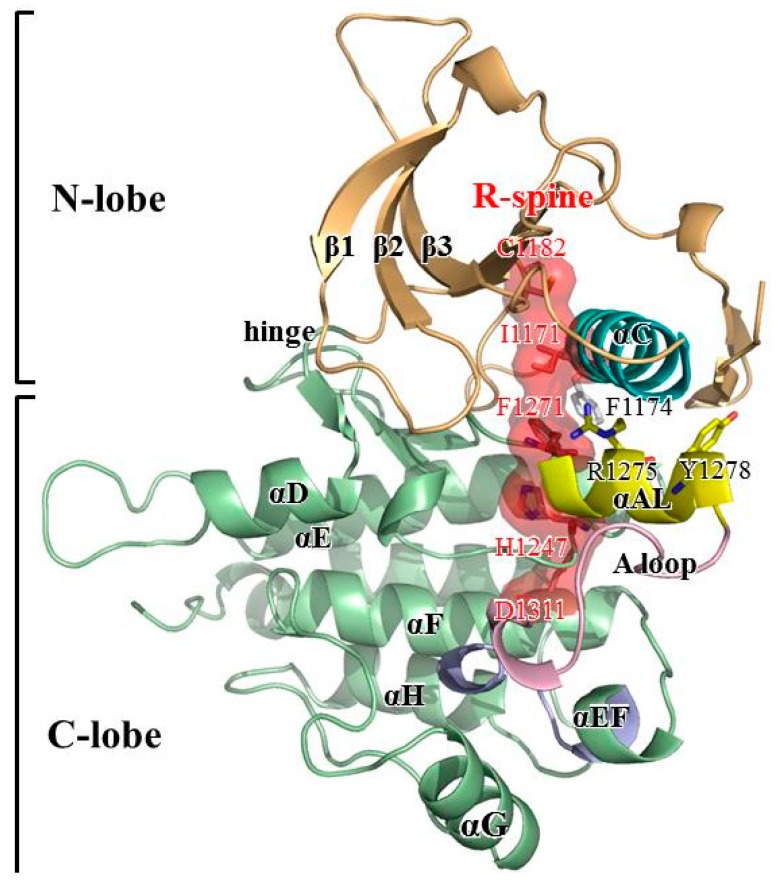
ALK structure retrieved from PDB 3L9P. Structural elements including the αC-helix, αAL-helix, A-loop, P+1 loop and components of the R-spine are highlighted. Residues to be altered including Y1278, F1174 and R1275 are also highlighted. Color code: orange for N-lobe, green for C-lobe, yellow for αAL-helix, blue for αC-helix, and red for R-spine.

Figure 1 presents an overview of the structure of the apo and inactive ALK protein kinase domain retrieved from the Protein Data Bank (PDB code: 3L9P) [8]. ALK is a typical protein kinase whose kinase domain consists of two lobes: the N-terminal small lobe (N-lobe) and C-terminal large lobe (C-lobe). The N-lobe includes one α-helix (αC-helix, residues 1158–1173) and five β-strands that form a relatively rigid antiparallel β-sheet. The C-lobe is mainly composed of helices with flexible loops. Between the two lobes is a cleft to accommodate adenosine triphosphate (ATP). In its active form, the N-lobe can move toward the C-lobe, whereas in its inactive form, the N-lobe is dynamically rigid and unable to take in ATP [9]. The ALK domain is autoinhibited because the JM segment (residues 1096–1103) folds in a β-turn motif and C1097′s amino hydrogen atom forms a hydrogen bond with the Y1278 hydroxy group, thereby prohibiting phosphorylation on Y1278. The A-loop structure also has an inhibitory arrangement; a short fragment of the proximal A-loop (the so-called αAL-helix (residues 1272–1280)) is packed beneath the αC-helix and accordingly prevents ALK from relaxing to its active conformation. Moreover, the distal portion of the A-loop (residues 1281–1291) is packed against the C-lobe, which blocks the P+1 pocket formed by the P+1 loop (residues 1292–1300, right after the A-loop’s C-terminus); thus, the P+1 pocket cannot accommodate the residues next to the residue to be phosphorylated by ALK [10,11,12,13].

Many fingerprint features distinguish the active and inactive structures of a kinase. For example, in an active state, the conserved DFG motif adopts a DFG-in with the phenylalanine pointing inward, which causes the ATP site to become available; by contrast, in an inactive state, the conserved DFG motif adopts a DFG-out position where the phenylalanine points outward and obstructs the ATP site [14,15,16]. In an active state, a salt bridge forms between a conserved glutamine on the αC-helix and a conserved lysine on the β3-strand and secures αC-helix orientation so that the relative motion between the N-lobe and C-lobe can favor the active state [17,18,19]. The assembled regulatory-spine (R-spine) is another hallmark of the active state [15,18,20,21]. Although the ALK domain in its basal condition is in an inactive state, it possesses several structural features of the active state. For example, the DFG motif adopts a DFG-in conformation, where F1271 points inward to make space for ATP binding; the salt bridge between E1161 on the αC-helix and R1192 on the β4-strand is formed so that the αC-helix orients perpendicularly to the αAL-helix. Moreover, the hydrophobic R-spine composed of C1182 (on the β4-strand), I1171 (on the βC-strand), F1271 (on the DFG motif, but only under the circumstance of DFG-in conformation), H1247 (on the HRD motif) and D1311 (on the αF-helix) is present in this inactive state structure. Because of these structural features, the ALK domain is a highly unique intermediate conformation between the active state (especially in the N-lobe region) and inactive state [15,18,20,21].

Mutations within the ALK domain that promote constitutive, ligand-independent activation are frequently involved in many diseases. Three “hot spot” residues are reportedly involved in 85% of all mutations, namely R1275 (43%), F1174 (30%) and F1245 (12%), where glutamine or leucine is typically substituted for R1275; leucine, isoleucine, valine, cysteine or serine replace F1174; and F1245 is replaced with leucine, isoleucine, valine or cysteine [22,23]. Experimental data have indicated that both F1174L and R1275Q transform ALK into a ligand-binding independent active form, evidenced by the rate constant *k*_cat_ (in min^−1^) values of 9.32 ± 0.85 for the wild type (WT), 119 ± 13 for R1275Q, 365 ± 61 for F1174L and 425 ± 63 for pY1278 [24]. In this study, we were interested in F1174L and R1275Q, which are commonly present in patients with neuroblastoma, a childhood cancer [1,25,26]. Figure 1 illustrates that F1174 is at the C-terminal end of the αC-helix and R1275 is situated at the middle of the αAL-helix. Molecular dynamics (MD) simulation has served as a useful tool to correlate protein structure and function at the atomic level [27,28,29,30,31]. In the present study, MD simulations were conducted to evaluate four ALK systems, namely WT, the F1174L variant, the R1275Q variant and phosphorylated WT (the pY1278 variant). Of these, WT is an inactive form, and the other three are active forms; we studied pY1278 to identify the common structural characteristics of the active form in the F1174L and R1275Q variants that correspond to the pY1278 variant. With this study, we wish to link the point mutation to the varied kinase function.

## 2. Materials and Methods

### System Setup

**Figure 2:**
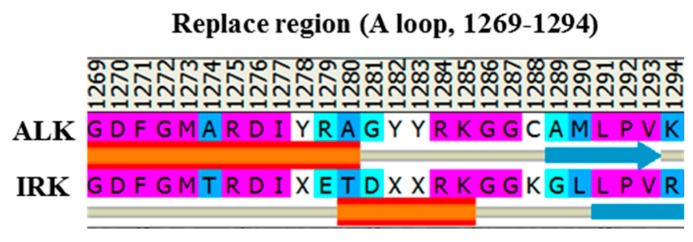
The sequence alignment of the IRK sequence against the target ALK sequence, where X stands for a phosphorylated tyrosine.

Three available crystal structures of the ALK, namely apo WT (PDB code: 3L9P) [8], the F1174L variant (PDB code: 4FNW) [25] and the R1275Q variant (PDB code: 4FNX) [25], were retrieved to serve as the initial structures for MD simulation to determine a possible structural discrepancy responsible for varied kinase activity. To construct the initial structure for the pY1278 variant, we used the aforementioned inactive apo WT structure (PDB code: 3L9P) for the structural base and replaced the A-loop fragment through homology modeling [32], which disclosed an A-loop from an active form structure of the insulin receptor tyrosine kinase (IRK, PDB code: 1IR3) [10]. Because both ALK and IRK belong to the insulin receptor kinase superfamily with the YXXXYY motif for autophosphorylation in their A-loops, we used the IRK structure, sharing a sequence identity of 41.3% with the ALK sequence, for A-loop homology. The sequence alignment of the IRK sequence against the target ALK sequence is shown in Figure 2. We then phosphorylated Y1278 on the replaced A-loop. The MD simulations were performed using the AMBER 12.0 software package [33,34] with ff03.r1 [35] and ff99SB [36,37] force fields. All hydrogen atoms of the four ALK systems were assigned using the LEaP module, which considered ionizable residues set at their default protonation states at a neutral pH value. Each studied ALK system was immersed in a cubic box of the TIP3P water model (10 Å minimum solute-wall distance), and five, six, five and six Na^+^ ions were added to neutralize WT, the pY1278 variant, the F1174L variant and the R1275Q variant, respectively. Each solvated ALK system underwent three stages of energy minimization; each stage consisted of 5000 steps of the steepest descent algorithm and 5000 steps of the conjugate gradient algorithm with a non-bonded cutoff of 8.0 Å. In Stage 1, all atoms in the ALK domain were restrained, thereby enabling the added TIP3P water molecules to reorient. In Stage 2, atoms in the protein backbone were restrained, and thus, the atoms in the amino acid side chains were rendered to interact with the added water molecules. In Stage 3, the whole solvated system was minimized without restraint to minimize conformational conflict.

The MD simulations in this study were performed in accordance with the standard protocol, which specifies gradual heating, density equilibration, equilibration and production procedures in the isothermal isobaric ensemble (NPT, P = 1 atm and T = 300 K) MD. A minimized solvated system was used as the starting structure in subsequent MD simulations. In the 100-ps heating procedure, the system was gradually heated from 0–300 K within 40 ps; this was followed by density equilibration at 300 K for 100 ps and then constant equilibration at 300 K for 1000 ps. Following the equilibration procedure, each complex system underwent two independent 100-ns production runs at a 2-fs time step. We recorded a snapshot every 10 ps throughout the production runs. An 8 Å cutoff was applied to treat nonbonding interactions such as short-range electrostatics and van der Waals interactions; the particle-mesh-Ewald method [38] was applied to treat long-range electrostatic interactions; and the SHAKE algorithm [39,40] was used to constrain all bonds containing hydrogen atoms to their equilibrium lengths. For structural and energetic analysis, we used the trajectory in the last 50 ns of each 100 ns MD run, which covered 200 × 2 = 400 conformation snapshots for each complex system.

## 3. Results and Discussion

### 3.1. MD Stability

**Figure 3:**
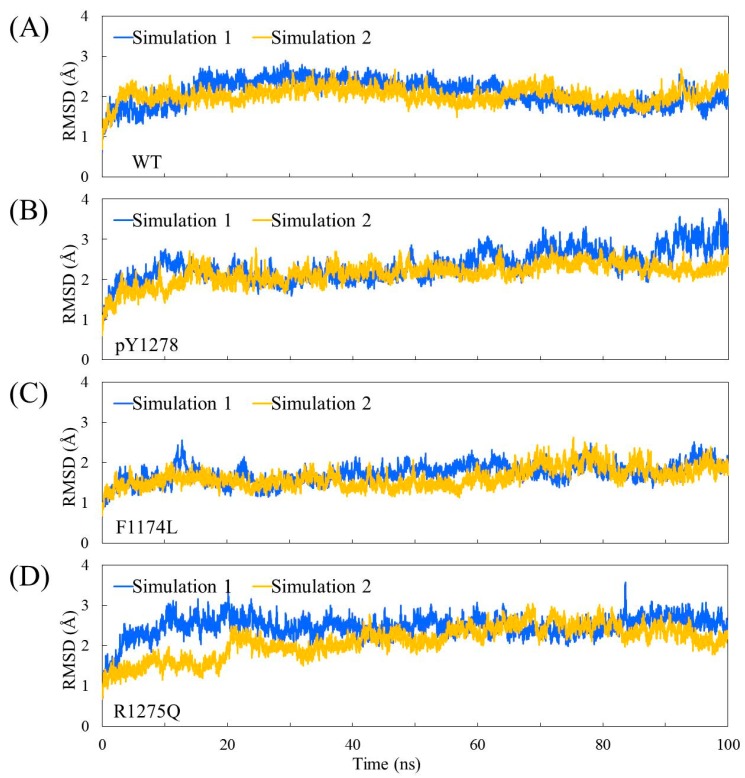
Time evolution of Cα RMSD values for two independent MD simulations of four studied ALK systems: (**A**) WT; (**B**) pY1278 variant; (**C**) F1174L variant and (**D**) R1275Q variant.

**Figure 4:**
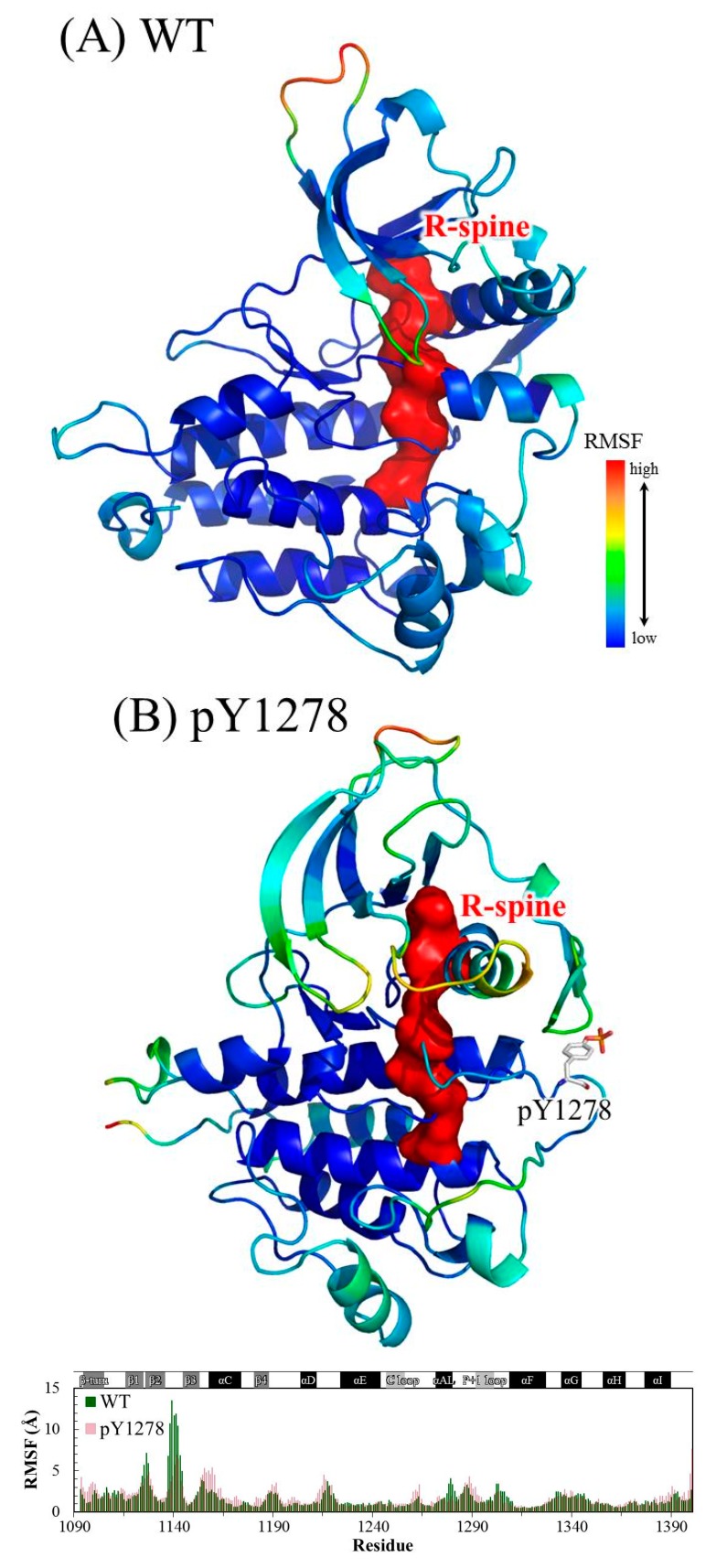

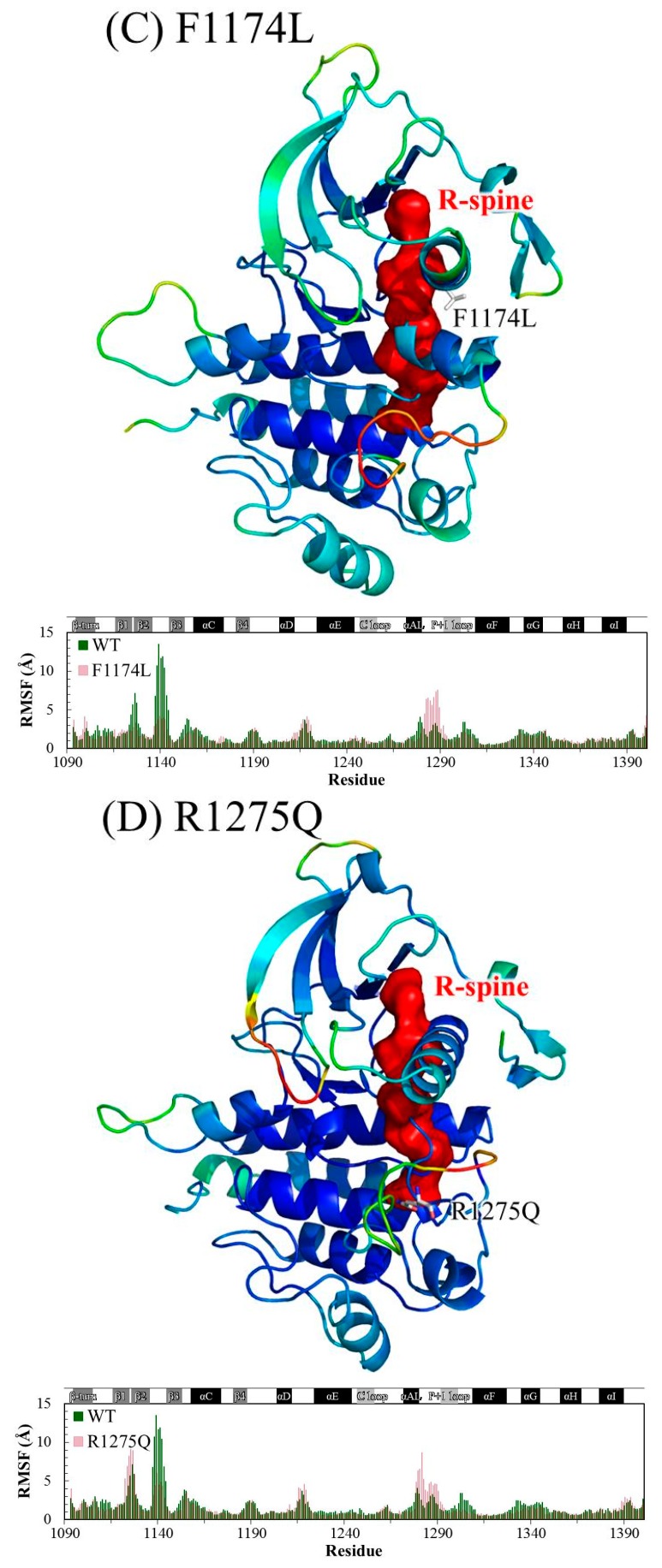
RMSF magnitudes of Cα atoms plotted on ALK structures of (**A**) WT; (**B**) pY1278 variant; (**C**) F1174L variant and (**D**) R1275Q variant. High to low mobility is denoted according to the red to blue color scale. Also shown in the bottom of each mutant panel is the RMSF value of each amino acid plotted against the WT. Color code: from blue to red for low to high relative RMSF.

The Cα root-mean-square deviation (RMSD) values for the four studied ALK systems, each system undergoing two independent 100 ns simulation runs, in the production duration given as a function of time are plotted in Figure 3; these values were used to monitor simulation trajectory quality and convergence. The curve of pY1278 Simulation 1 fluctuated at nearly a 2 Å magnitude (with a minimum of 1.8 Å at 71 ns and a maximum of 3.8 Å at 98 ns), whereas the other seven curves fluctuated at a minor magnitude of 1 Å variance. For each studied ALK system, we collected 400 conformations from the two independent MD simulation runs (200 conformations from one MD trajectory within 50–100 ns) and conducted structural and dynamic analysis. Using the 400 collected conformations, the root-mean-square fluctuation (RMSF) per amino acid residue was gauged and plotted over the structure shown in Figure 4. As evident in Figure 4A, concerning WT, the most flexible regions were the β2–β3 loop (scaled in red) and the P-loop (between β1 and β2, scaled in greenish yellow). Notably, the β2–β3 loop was too flexible to be determined in the crystallography solved structure; therefore, the high mobility in the modeled β2–β3 loop was expected. Furthermore, the low mobility on the αC-helix, αAL-helix and distal A-loop was anticipated to maintain ALK inactivity. As depicted in Figure 4B, the pY1278 variant demonstrated notable mobility on the β2–β3 loop (scaled in red), P-loop (scaled in yellow), N-terminal end of the αC-helix (scaled in orange and yellow) and P+1 loop (scaled in green). Figure 4C illustrates that the αAL-helix remained structured despite the F1174L mutation; however, the distal A-loop exhibited high mobility all the way to the P+1 loop, as indicated by the red fragment in the figure. Figure 4D illustrates how the A-loop in the R1275Q variant was entirely destructured and mobile. Moreover, the four residues encompassed by the continuous red surfaces in Figure 4A–D indicate that all four studied ALK systems had assembled R-spines, as mentioned in the Introduction section. Counted from the N-lobe to the C-lobe, the third component of the R-spine was F1271 in the DFG motif only under the condition that the DFG motif adopted a DFG-in conformation, and the pointing-inward F1271 participated in R-spine formation and enforced R-spine assembly.

### 3.2. Structural Variance on the A-loop

**Figure 5:**
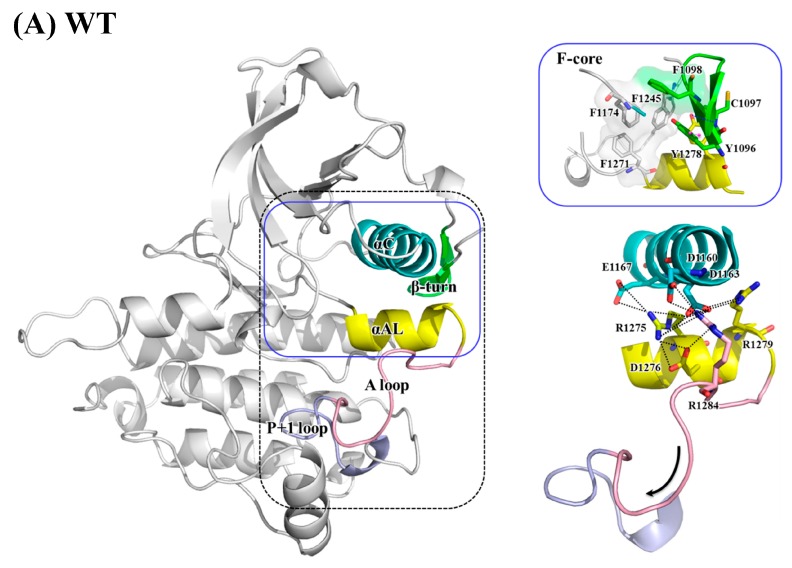

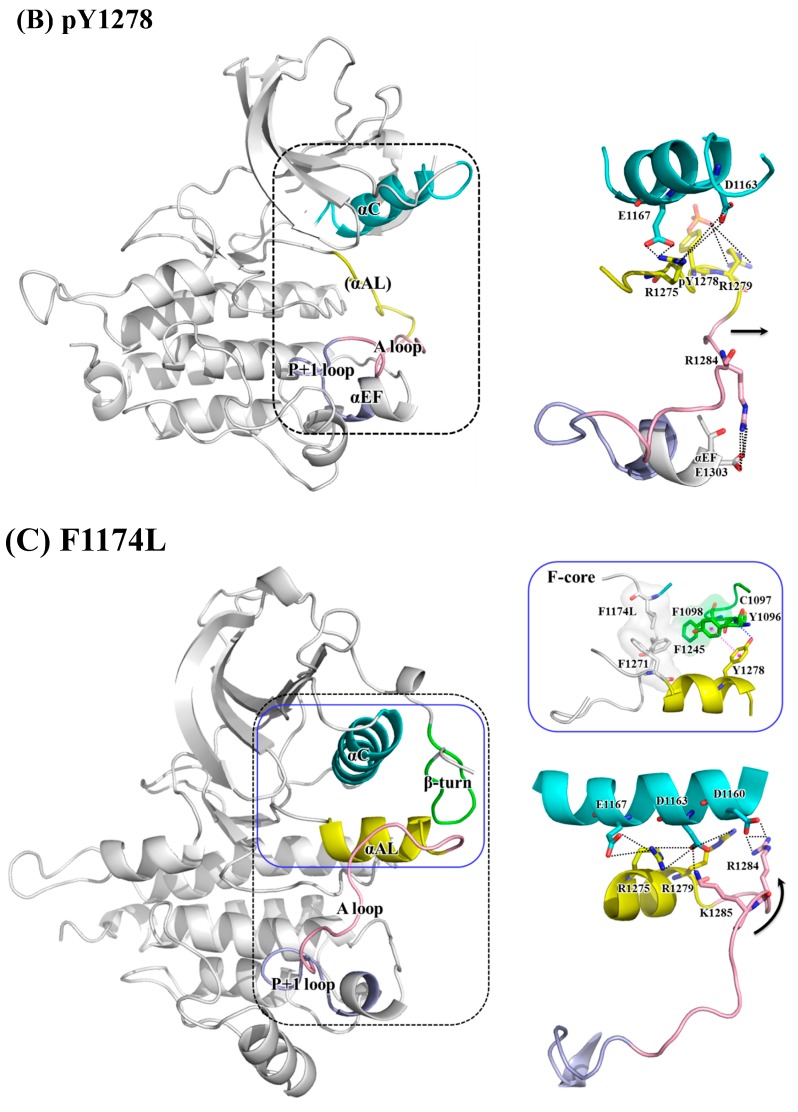

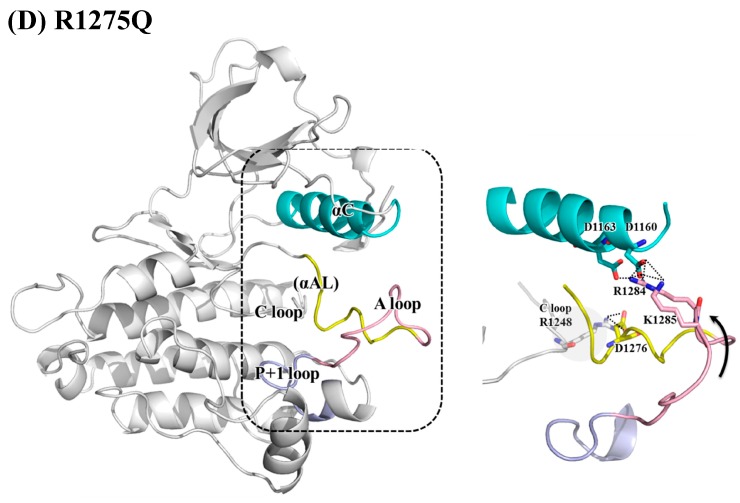
Main interactions and structural variance from the αC-helix, αAL-helix, A-loop and P+1 loop in (**A**) WT; (**B**) pY1278 variant; (**C**) F1174L variant; and (**D**) R1275Q variant. Color code: pink for A-loop, yellow for αAL-helix, and blue for αC-helix.

Figure 5 displays the electrostatic interactions centered at the A-loop and its nearby regions, namely the αC-helix and P+1 loop. Regarding WT ALK, Figure 5A presents dense electrostatic interactions between the αC- and αAL-helices to anchor the αAL-helix. D1160, D1163 and E1167 on the αC-helix interacted with R1275, R1279 and R1284 on the αAL-helix. The hydrogen bond between Y1278 and C1097 (on the N-terminal β-turn) and π–π interaction between Y1278 and Y1096 (also on the N-terminal β-turn) also stabilized the αAL-helix. Additionally, the salt bridge formed by the adjacent R1275 and D1276 stabilized the αAL-helix structure. R1284 on the distal A-loop was anchored by D1163 on the αC-helix and D1276 on the αAL-helix, and thus, the distal A-loop packed against the C-lobe and sat above the P+1 loop, thereby blocking the P+1 pocket for peptide substrate binding.

As shown in Figure 5B, which illustrates the pY1278 variant, the electronegative phosphate group on Y1278 released the linkage between the A-loop and N-terminal β turn and generated a new linkage toward the adjacent R1279 that was used to form salt bridges with the αC-helix in WT. The rearrangement of Y1278 and R1279 unfolded the αAL-helix and consequently interrupted the aforementioned electrostatic contact between the αC- and αAL-helices, thereby causing the A-loop to move backward rather than to sit above the P+1 loop. Furthermore, a newly-formed salt bridge between R1284 (on the A-loop) and E1303 (on the αEF-helix) played an auxiliary role in fixing the C-terminal portion of the A-loop and significantly exposed the P+1 pocket.

Figure 5C for the F1174L variant shows that the αAL-helix structure still held, as did the interaction between the αAL-helix and αC-helix. Lee et al. found that in WT, a hydrophobic F-core was formed by F1174 (on the C-terminal αC-helix), F1098 (on the N-terminal β-turn), F1271 (on the DFG motif) and F1245 (on the C-loop); when the smaller residue, namely leucine, replaced F1174, the F-core was maintained [41]. Our structural analysis suggested that the F-core became relatively compact because of the smaller L1174; this structural compactness drew the distal A-loop upward, as evidenced by the enhanced salt bridges between R1284 and K1285 (both on the distal A-loop) toward D1160 (on the αC-helix) and D1163 (on the αC-helix), and consequently moved the A-loop upward, as well. Together, these conformational changes rendered the P+1 pocket accessible.

As discussed, R1275 on the αAL-helix plays a dual role, namely securing the electrostatic interactions between the αC- and αAL-helices and preserving the helical conformation of the αAL-helix through the R1275–D1276 interaction. In the R1275Q variant displayed in Figure 5D, the substituted Q1275 no longer linked to D1276, and the detached D1276 moved backward and formed a salt bridge with R1248 on the C-loop. The αAL-helix was distorted and lifted the electropositive R1284 and R1285 to interact with the electronegative D1160 and D1163 on the αC-helix. This upward movement also elevated the A-loop and revealed the P+1 pocket. The conformations that can show the abovementioned interactions were chosen for Figure 5.

## 4. Conclusions

**Figure 6:**
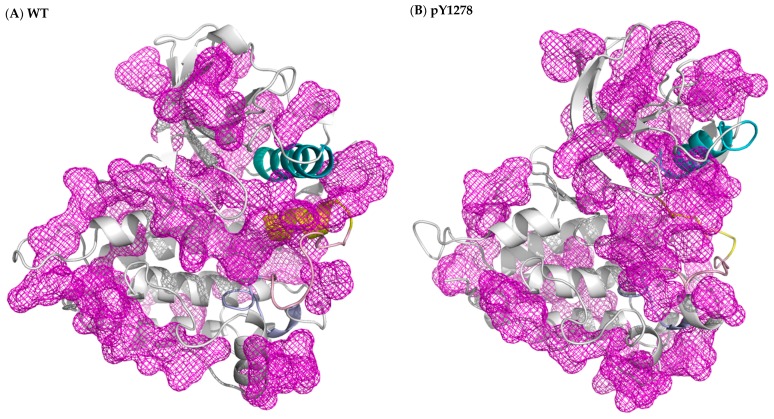

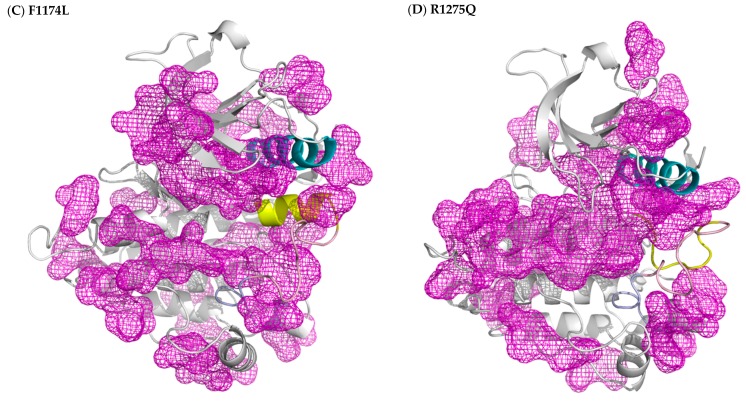
Protein cavity detection achieved using fpocket on (**A**) WT, (**B**) pY1278 variant, (**C**) F1174L variant and (**D**) R1275Q variant. The purple regions in mesh are predicted grooves for substrate binding. The cavity is searched by a probe, in terms of the alpha sphere, screening along the protein surface. An alpha sphere is a contact sphere that touches four atoms in 3D space without any internal atoms. As for the fpocket parameter setting, the alpha sphere radius was set as 3 and 7 Å for the minimum and maximum, and a cavity must contain at least 25 spheres. Color code: blue for αC-helix, yellow for intact or disrupted αAL-helix, and pink for A loop.

In this MD simulation study, we used the X-ray crystal structures of ALK in its WT, F1174L and R1275Q variants to elucidate the activation mechanism of the F1174L and R1275Q mutations. We also generated a structure of WT-carrying phosphorylated Y1278 to obtain structural features of the active ALK. The DFG motif in each of the four studied ALK systems adopted the DFG-in conformation, and thus, the DFG phenylalanine was able to participate in R-spine assembly regardless of the inactive state of WT ALK. Both DFG-in and the presence of R-spine were characteristic of the active state of kinases. The WT ALK structure was known to be an intermediate between the active and inactive states. Because of the DFG-in conformation, the DFG phenylalanine pointed inward and rendered the ATP binding site available, as in active kinase structures. However, the N-terminal portion of the A-loop folded as the αAL-helix remained in close contact with the αC-helix and gradually influenced the spatial arrangement of the C-terminal portion of the A-loop to seal the P+1 pocket generated by the P+1 loop. With respect to the pY1275, F1174L and R1275Q variants, the A-loop moved either upward or to the right and accordingly exposed the P+1 pocket for the substrate. In summary, we conducted protein cavity detection using fpocket [42] and plotted the results in Figure 6, where the regions in mesh represent cavities in the protein. In all four studied ALK systems, the ATP binding sites were attainable; however, in contrast to the three variants, WT’s P+1 pocket for the peptide substrate was occluded by the C-terminal portion of the A-loop. The ATP site was ready for processing phosphorylation in WT ALK, but the peptide substrate binding site was not yet available. Our results provide the key insight that regulation on the P+1 pocket plays an equally decisive role, as does the ATP pocket in the activation mechanism in kinases.

## References

[1] Roskoski R. (2013). Anaplastic lymphoma kinase (ALK): Structure, oncogenic activation, and pharmacological inhibition. Pharmacol. Res..

[2] Salaverria I., Beà S., Lopez-Guillermo A., Lespinet V., Pinyol M., Burkhardt B., Lamant L., Zettl A., Horsman D., Gascoyne R. (2008). Genomic profiling reveals different genetic aberrations in systemic ALK-positive and ALK-negative anaplastic large cell lymphomas. Br. J. Haematol..

[3] Katayama R. (2018). Drug resistance in anaplastic lymphoma kinase-rearranged lung cancer. Cancer Sci..

[4] Chen Y., Takita J., Choi Y.L., Kato M., Ohira M., Sanada M., Wang L., Soda M., Kikuchi A., Igarashi T. (2008). Oncogenic mutations of ALK kinase in neuroblastoma. Nature.

[5] Stoica G.E., Kuo A., Powers C., Bowden E.T., Sale E.B., Riegel A.T., Wellstein A. (2002). Midkine binds to anaplastic lymphoma kinase (ALK) and acts as a growth factor for different cell types. J. Biol. Chem..

[6] Stoica G.E., Kuo A., Aigner A., Sunitha I., Souttou B., Malerczyk C., Caughey D.J., Wen D., Karavanov A., Riegel A.T. (2001). Identification of anaplastic lymphoma kinase as a receptor for the growth factor pleiotrophin. J. Biol. Chem..

[7] Tartari C.J., Gunby R.H., Coluccia A.M., Sottocornola R., Cimbro B., Scapozza L., Donella-Deana A., Pinna L.A., Gambacorti-Passerini C. (2008). Characterization of some molecular mechanisms governing autoactivation of the catalytic domain of the anaplastic lymphoma kinase. J. Biol. Chem..

[8] Lee C.C., Jia Y., Li N., Sun X., Ng K., Ambing E., Gao M.-Y., Hua S., Chen C., Kim S. (2010). Crystal structure of the ALK (anaplastic lymphoma kinase) catalytic domain. Biochem. J..

[9] Chen H., Ma J., Li W., Eliseenkova A.V., Xu C., Neubert T.A., Miller W.T., Mohammadi M. (2007). A molecular brake in the kinase hinge region regulates the activity of receptor tyrosine kinases. Mol. Cell.

[10] Hubbard S.R. (1997). Crystal structure of the activated insulin receptor tyrosine kinase in complex with peptide substrate and ATP analog. EMBO J..

[11] Songyang Z., Lu K.P., Kwon Y.T., Tsai L.-H., Filhol O., Cochet C., Brickey D.A., Soderling T.R., Bartleson C., Graves D.J. (1996). A structural basis for substrate specificities of protein Ser/Thr kinases: Primary sequence preference of casein kinases I and II, NIMA, phosphorylase kinase, calmodulin-dependent kinase II, CDK5, and Erk1. Mol. Cell. Biol..

[12] Songyang Z., Blechner S., Hoagland N., Hoekstra M.F., Piwnica-Worms H., Cantley L.C. (1994). Use of an oriented peptide library to determine the optimal substrates of protein kinases. Curr. Biol..

[13] Shoelson S.E., Chatterjee S., Chaudhuri M., White M.F. (1992). YMXM motifs of IRS-1 define substrate specificity of the insulin receptor kinase. Proc. Natl. Acad. Sci. USA.

[14] Yang J., Cron P., Thompson V., Good V.M., Hess D., Hemmings B.A., Barford D. (2002). Molecular mechanism for the regulation of protein kinase B/Akt by hydrophobic motif phosphorylation. Mol. Cell.

[15] Taylor S.S., Kornev A.P. (2011). Protein kinases: Evolution of dynamic regulatory proteins. Trends Biochem. Sci..

[16] Kornev A.P., Haste N.M., Taylor S.S., Ten Eyck L.F. (2006). Surface comparison of active and inactive protein kinases identifies a conserved activation mechanism. Proc. Natl. Acad. Sci. USA.

[17] Palmieri L., Rastelli G. (2013). αC helix displacement as a general approach for allosteric modulation of protein kinases. Drug Discov. Today.

[18] Kornev A.P., Taylor S.S. (2010). Defining the conserved internal architecture of a protein kinase. BBA Proteins Proteom..

[19] Till J.H., Becerra M., Watty A., Lu Y., Ma Y., Neubert T.A., Burden S.J., Hubbard S.R. (2002). Crystal structure of the MuSK tyrosine kinase: Insights into receptor autoregulation. Structure.

[20] Taylor S.S., Zhang P., Steichen J.M., Keshwani M.M., Kornev A.P. (2013). PKA: Lessons learned after twenty years. BBA Proteins Proteom..

[21] Roskoski R. (2012). ERK1/2 MAP kinases: Structure, function, and regulation. Pharmacol. Res..

[22] Bresler S.C., Weiser D.A., Huwe P.J., Park J.H., Krytska K., Ryles H., Laudenslager M., Rappaport E.F., Wood A.C., McGrady P.W. (2014). ALK mutations confer differential oncogenic activation and sensitivity to ALK inhibition therapy in neuroblastoma. Cancer Cell.

[23] Hallberg B., Palmer R.H. (2013). Mechanistic insight into ALK receptor tyrosine kinase in human cancer biology. Nat. Rev. Cancer.

[24] Bresler S.C., Wood A.C., Haglund E.A., Courtright J., Belcastro L.T., Plegaria J.S., Cole K., Toporovskaya Y., Zhao H., Carpenter E.L. (2011). Differential inhibitor sensitivity of anaplastic lymphoma kinase variants found in neuroblastoma. Sci. Transl. Med..

[25] Epstein L.F., Chen H., Emkey R., Whittington D.A. (2012). The R1275Q neuroblastoma mutant and certain ATP-competitive inhibitors stabilize alternative activation loop conformations of anaplastic lymphoma kinase. J. Biol. Chem..

[26] Berry T., Luther W., Bhatnagar N., Jamin Y., Poon E., Sanda T., Pei D., Sharma B., Vetharoy W.R., Hallsworth A. (2012). The ALKF1174L mutation potentiates the oncogenic activity of MYCN in neuroblastoma. Cancer Cell.

[27] Fischer N., Neumann P., Konevega A.L., Bock L.V., Ficner R., Rodnina M.V., Stark H. (2015). Structure of the E. coli ribosome-EF-Tu complex at <3 Å resolution by C s-corrected cryo-EM. Nature.

[28] Grouleff J., Irudayam S.J., Skeby K.K., Schiøtt B. (2015). The influence of cholesterol on membrane protein structure, function, and dynamics studied by molecular dynamics simulations. BBA Proteins Proteom..

[29] Miao Y., Caliman A.D., McCammon J.A. (2015). Allosteric effects of sodium ion binding on activation of the M3 muscarinic G-protein-coupled receptor. Biophys. J..

[30] Sborgi L., Verma A., Piana S., Lindorff-Larsen K., Cerminara M., Santiveri C.M., Shaw D.E., De Alba E., Munñoz V. (2015). Interaction networks in protein folding via atomic-resolution experiments and long-time-scale molecular dynamics simulations. J. Am. Chem. Soc..

[31] Khan F.I., Govender A., Permaul K., Singh S., Bisetty K. (2015). Thermostable chitinase II from Thermomyces lanuginosus SSBP: Cloning, structure prediction and molecular dynamics simulations. JTBio.

[32] Lee C.C., Chuang Y.C., Liu Y.L., Yang C.N. (2017). A molecular dynamics simulation study for variant drug responses due to FMS-like tyrosine kinase 3 G697R mutation. RSC Adv..

[33] Salomon-Ferrer R., Case D.A., Walker R.C. (2012). An overview of the Amber biomolecular simulation package. Wiley Interdiscip. Rev. Comput. Mol. Sci..

[34] Case D., Darden T., Cheatham T., Simmerling C., Wang J., Duke R., Luo R., Walker R., Zhang W., Merz K. (2012). Assisted Model Building with Energy Refinement (AMBER) 12.

[35] Duan Y., Wu C., Chowdhury S., Lee M.C., Xiong G., Zhang W., Yang R., Cieplak P., Luo R., Lee T. (2003). A point-charge force field for molecular mechanics simulations of proteins based on condensed-phase quantum mechanical calculations. JCoCh.

[36] Cornell W.D., Cieplak P., Bayly C.I., Gould I.R., Merz K.M., Ferguson D.M., Spellmeyer D.C., Fox T., Caldwell J.W., Kollman P.A. (1995). A second generation force field for the simulation of proteins, nucleic acids, and organic molecules. J. Am. Chem. Soc..

[37] Hornak V., Abel R., Okur A., Strockbine B., Roitberg A., Simmerling C. (2006). Comparison of multiple Amber force fields and development of improved protein backbone parameters. Proteins Struct. Funct. Bioinform..

[38] Hockney R.W., Eastwood J.W. (1988). Computer Simulation Using Particles.

[39] Ryckaert J.-P., Ciccotti G., Berendsen H.J. (1977). Numerical integration of the cartesian equations of motion of a system with constraints: Molecular dynamics of n-alkanes. J. Comput. Phys..

[40] Van Gunsteren W., Berendsen H. (1977). Algorithms for macromolecular dynamics and constraint dynamics. Mol. Phys..

[41] Munson M., Balasubramanian S., Fleming K.G., Nagi A.D., O’Brien R., Sturtevant J.M., Regan L. (1996). What makes a protein a protein?. Hydrophobic core designs that specify stability and structural properties. Protein Sci..

[42] Le Guilloux V., Schmidtke P., Tuffery P. (2009). Fpocket: An open source platform for ligand pocket detection. BMC Bioinform..

